# Eccrine Sweat Contains IL-1α, IL-1β and IL-31 and Activates Epidermal Keratinocytes as a Danger Signal

**DOI:** 10.1371/journal.pone.0067666

**Published:** 2013-07-11

**Authors:** Xiuju Dai, Hidenori Okazaki, Yasushi Hanakawa, Masamoto Murakami, Mikiko Tohyama, Yuji Shirakata, Koji Sayama

**Affiliations:** Department of Dermatology, Ehime University Graduate School of Medicine, Toon, Ehime, Japan; CNRS-University of Toulouse, France

## Abstract

Eccrine sweat is secreted onto the skin's surface and is not harmful to normal skin, but can exacerbate eczematous lesions in atopic dermatitis. Although eccrine sweat contains a number of minerals, proteins, and proteolytic enzymes, how it causes skin inflammation is not clear. We hypothesized that it stimulates keratinocytes directly, as a danger signal. Eccrine sweat was collected from the arms of healthy volunteers after exercise, and levels of proinflammatory cytokines in the sweat were quantified by ELISA. We detected the presence of IL-1α, IL-1β, and high levels of IL-31 in sweat samples. To investigate whether sweat activates keratinocytes, normal human keratinocytes were stimulated with concentrated sweat. Western blot analysis demonstrated the activation of NF-κB, ERK, and JNK signaling in sweat-stimulated keratinocytes. Real-time PCR using total RNA and ELISA analysis of supernatants showed the upregulation of IL-8 and IL-1β by sweat. Furthermore, pretreatment with IL-1R antagonist blocked sweat-stimulated cytokine production and signal activation, indicating that bioactive IL-1 is a major factor in the activation of keratinocytes by sweat. Moreover, IL-31 seems to be another sweat stimulator that activates keratinocytes to produce inflammatory cytokine, CCL2. Sweat is secreted onto the skin's surface and does not come into contact with keratinocytes in normal skin. However, in skin with a defective cutaneous barrier, such as atopic dermatitis-affected skin, sweat cytokines can directly act on epidermal keratinocytes, resulting in their activation. In conclusion, eccrine sweat contains proinflammatory cytokines, IL-1 and IL-31, and activates epidermal keratinocytes as a danger signal.

## Introduction

Skin epithelial cells that interact with various external stimuli produce cytokines and chemokines to initiate immune responses. Sweat is secreted by eccrine glands onto the skin's surface, which is a physiological process and necessary for thermoregulation. Despite being not harmful to normal skin, sweat acts as a common aggravating factor in development of atopic dermatitis (AD), a chronic inflammatory skin disease involving gene-environment interaction. And dealing with sweating is important to prevent itching and aggravation of AD in summer [1]. While AD patients often exhibit a defective ability to sweat in response to thermal stress in affected skin, marked augmentation of the sweating response with delayed kinetics can be paradoxically detected in some eccrine glands [2], [3], indicating compensatory hyperhidrosis, which may contribute to the exacerbation of AD lesions. Abnormalities in the transport of sweat onto the skin's surface, resulting in the intra-epidermal retention of sweat, can cause a severe prickly sensation and skin inflammation, as exemplified by miliaria rubra [4]. Miliaria rubra, with obstruction and rupture of intra-epidermal eccrine ducts, is characterized by spongiotic vesicles in the stratum malpighii and chronic inflammation around the dermal ducts [4]. While sweat is involved in the exacerbation of itchy chronic inflammatory dermatitis, little attention has been paid to the beneficial role of sweat in the development of skin inflammation.

In normal sweat obtained from healthy volunteers, in addition to proteolytic enzymes, antigens, and histamine, anti-microbial peptides [5] and the proinflammatory cytokines interleukin (IL)-1 and IL-8 [6]–[8] have been quantified. As sweat is secreted onto the skin's surface, there is no direct contact between sweat and keratinocytes in healthy skin, so it is unlikely to have a physiological role in keratinocyte activation. However, in AD-affected skin with a defective epidermal barrier, or in skin affected by miliaria rubra, which is characterized by the rupture of intra-epidermal eccrine glands, sweat materials will penetrate into the epidermis, come into contact with keratinocytes, and stimulate their activation, as sensitizers. In this study, we showed that eccrine sweat directly activates epidermal keratinocytes and that this inflammatory function of sweat is dependent on the bioactivity of sweat IL-1 and IL-31.

## Materials and Methods

### Ethics Statement

This study was performed according to the principles set forth in the Declaration of Helsinki. All procedures involving human subjects received prior approval from the ethical committee of Ehime University School of Medicine. The volunteers, patients involved in the study, and the guardians on behalf of children participants provided written informed consent before experiments were initiated.

### Reagents

The antibodies for inhibitor κB α (IκBα), phospho-IκBα, p38, phospho-p38, extracellular signal-regulated kinase (ERK), phospho-ERK, c-Jun N-terminal kinase (JNK), phospho-JNK, signal transducer and activator of transcription (STAT)3 and phospho-STAT3, were obtained from Cell Signaling Technology, Inc. (Beverly, MA, USA); and anti-β-actin was purchased from Santa Cruz Biotechnology (Santa Cruz, CA, USA). Human recombinant cytokines, IL-31, IFN-γ, IL-1β, IL-4 and IL-13, and the IL-1R antagonist (IL-1Ra) were obtained from R&D Systems (MN, USA) and dissolved according to the manufacturer's instructions.

### Keratinocyte culture and stimulation

Primary human keratinocytes were isolated from surgically discarded neonatal skin samples, and cultured in MCDB153 medium, supplemented with insulin (5 μg ml^−1^), hydrocortisone (5×10^−7^ M), ethanolamine (0.1 mM), phosphoethanolamine (0.1 mM), bovine hypothalamic extract (50 μg ml^−1^) and Ca^2+^ (0.03 mM), as described previously [9]. Cells that had been passaged four times were used in the experiments, and subconfluent keratinocyte cultures were subjected to stimulation.

### Sweat collection and processing

Using tissue paper, eccrine sweat was collected from the arms of 11 healthy volunteers (students of Ehime University; 3 women and 8 men; age, 19–23 years) after 30 min of exercise as described previously [5]. After collection, the crude sweat was centrifuged at 15,000 r.p.m. for 10 min, then supernatant collected and stored at −80°C until ELISA analysis. Total protein concentration of sweat samples was evaluated by Bio-Rad protein assay (Bio-Rad, CA) according to the manufacturer's instruction. For the experiment of cell stimulation, 3 ml of the crude sweat was centrifuged at 15,000 r.p.m. for 10 min, supernatant collected. After repeating once, the samples were desalted with Sephadex G-10 (GE Healthcare, Tokyo, Japan), lyophilized, then resuspended in distilled water and filtered through 0.45 um filter. To stimulate keratinocytes, concentrated sweats (at the concentration of 50 ng/ml) obtained from three healthy volunteers were added to cultures for a predetermined period, respectively.

### RNA preparation, real-time reverse transcription–polymerase chain reaction (RT–PCR) and RT-PCR

Total RNA was isolated using Isogen (Nippon Gene, Japan). Primers and probes specific for GAPDH (Glyceraldehyde-3-phosphate dehydrogenase), IL-1β, IL-8, RIG (retinoic acid-inducible gene-I), NOD2 (Nucleotide-binding oligomerization domain-containing protein 2) and CCL2 were obtained from Applied Biosystems. Real-time RT–PCR was performed using an ABI PRISM 7700 sequence detector (Applied Biosystems, NJ) according to the manufacturer's protocol. The mRNA expression of target genes was normalized to that of GAPDH. Levels of gene expression in treated cells were quantified relative to those in untreated cells. The mRNA expression of IL-31 receptor A (IL-31RA) and oncostatin M receptor (OSMR) was examined using the primer pairs as described previously [10]. RT–PCR was performed using RT–PCR High Plus (Toyobo, Osaka, Japan). The products were visualized on 2% agarose gels containing ethidium bromide and were then sequenced to confirm the accuracy of amplification.

### ELISA

Sweat samples obtained from healthy volunteers were prepared and culture supernatants were collected. ELISA kits for IL-1α, IL-1β, IL-8, and IL-31 were purchased from R&D Systems (Minneapolis, Minn). ELISAs were performed according to the manufacturer's protocol. The optical density at 450 nm was measured with an Immuno Mini NJ-2300 microplate reader (Nalgene Nunc International K.K., Tokyo, Japan). All assays were performed in triplicate.

### Protein preparation and western blotting

The cells were harvested by transferring them into an extraction buffer containing 150 mM NaCl, 1% NP-40, 0.5% deoxycholate, 0.1% SDS, 50 mM s–HCl (pH 7.4) and protease inhibitors. Equal amounts of protein were separated by SDS–PAGE and transferred onto polyvinylidene difluoride membranes. The analysis was performed using a Vistra ECF Kit (Amersham Biosciences K.K., Tokyo, Japan) and a FluoroImager (Molecular Dynamics Inc., Sunnyvale, CA, USA).

### Immunohistological staining

Biopsies from surgically discarded normal skin were fixed in formaldehyde, then embedded in paraffin and 4∼5 μm sections were prepared. For IL-31 staining, the fixed sections were deparaffinized and adapted for antigen retrieval. Mouse anti-IL-31 (R&D Systems) at 10 μg/ml was used. Staining was performed according to the manufacturer's instructions using ImmPRESS^TM^ detection system (Vector Laboratories, Inc., Burling, CA).

### Statistical analysis

At least three independent studies were performed and yielded similar results. The results of one representative experiment are shown in each of the figures. Quantitative data are expressed as means ± standard deviations. Statistical significance was determined using the paired Student's *t*-test. Differences were deemed statistically significant at *P*<0.05. The levels of statistical significance are indicated in the figures as follows: *P<0.05.

## Results

### The presence of IL-1α, IL-1β, and IL-31 in sweat from healthy volunteers

**Figure 1:**
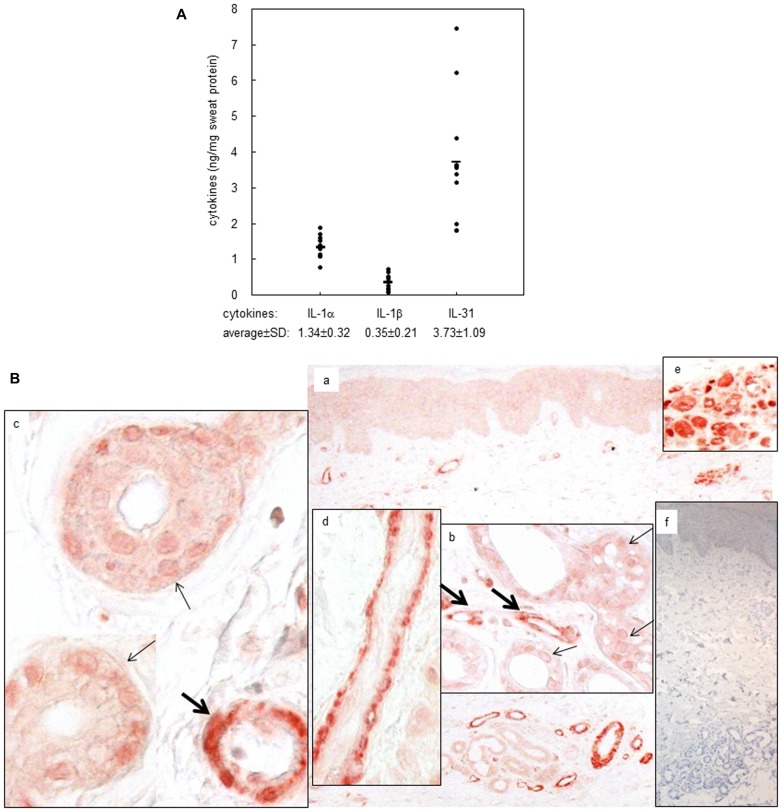
IL-1α, IL-1β and IL-31 were quantified in the sweats obtained from volunteers. (A) Sweat samples obtained from 11 healthy volunteers were subjected to ELISA, and the concentrations of IL-1α, IL-1β and IL-31 were detected and quantified against the levels of total sweat protein, respectively. Concentrations (expressed as ng/mg sweat protein) represent means±SD (*n* = 11). (B) Immunohistochemistry showed the specific expression of IL-31 protein in eccrinne gland in normal skin. (a) The specific staining of IL-31 protein in the eccrine gland apparatus (original magnification: 40X) (b) IL-31 staining in eccrine gland and duct (original magnification: 200X) (c) IL-31 staining in eccrine gland and duct (original magnification: 400X) (d) the marked IL-31 staining in eccrine straight duct (original magnification: 400X) (e) the marked IL-31 staining in the coiled eccrine ducts (original magnification: 200X) (f) negative staining with mouse IgG and then nuclear staining with haematoxylin (original magnification: 40X) (small arrows indicate eccrine glands, and big arrows indicate eccrine ducts).

We first evaluated the levels of proinflammatory cytokines in sweat obtained from healthy volunteers. Sweat samples collected from the arms of 11 volunteers were prepared and subjected to ELISA analysis to quantify the levels of IL-1α, IL-1β, TNF-α, IL-6, and IL-8, cytokines that are expressed by sweat gland cells [6], [7], [11]–[13]. Consistent with previous studies using sweat from palmoplantar regions of healthy volunteers [7], [8], all arm sweat samples were found to contain significant amounts of immunoreactive IL-1α and IL-1β, with average concentrations of 1.34 and 0.35 ng/mg sweat protein, respectively (Fig. 1A). However, no IL-6 or TNF-α was detected in any of the sweat samples, and only low levels of IL-8 were detected in three of 11 samples (data not shown).

Recently, IL-31, a novel Th2 cytokine, was linked to the pathogenesis of several pruritic skin diseases [14]. As sweat often causes itchiness in inflammatory lesions [4], we determined whether IL-31 is secreted together with sweat. As shown in Fig. 1A, IL-31 was detected in the sweat from healthy volunteers, with an average concentration of 3.73 ng/mg sweat protein, which is higher than the level of sweat IL-1. The *in situ* expression of IL-1α and IL-1β in normal human skin eccrine sweat gland ductal and secretory epithelium has been well reported previously [11]–[13]. To investigate the cellular source of sweat IL-31, we performed immunohistochemical analysis of normal skin to assess whether IL-31is *in situ* expressed by eccrine gland cells. As shown in Fig. 1B, compared to other skin apparatuses, the eccrine gland structure (both the gland and the duct) was strongly stained with anti-IL-31 antibody (Fig. 1B). In spite of its relatively weak labeling in the secretory cells of eccrine glands, IL-31 was strongly detected in the epithelium of coiled and straight ducts, with more intense labeling in the basal cells than that in the inner luminal cells (Fig. 1B). In summary, we quantified IL-1α, IL-1β, and IL-31 in normal sweat and demonstrated the in situ expression of IL-31 in eccrine gland cells.

### Sweat stimulates the activation of cultured normal human keratinocytes

**Figure 2:**
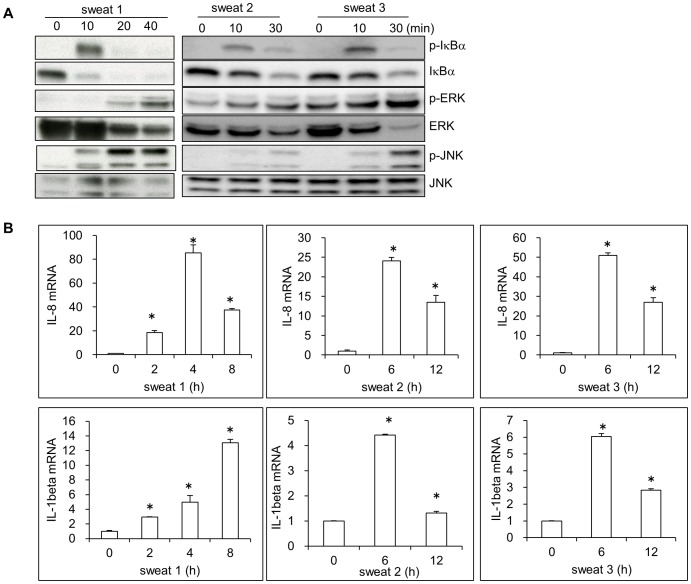
Sweat stimulated signal activation and induced cytokine production in keratinocytes. (A) Sweats obtained from three volunteers were respectively concentrated and added into cultures (50 ng/ml), the levels of IκBα, phospho-IκBα, ERK, phospho-ERK, JNK and phospho-JNK in keratinocytes were examined by Western blotting. (B) Keratinocytes were stimulated with concentrated sweat, and the expression of IL-8 and IL-1β mRNA was detected by real time PCR. The relative mRNA levels are expressed as means±SD (*n* = 3). *p<0.05: compared to that of 0 h.

Considering the presence of cytokines in sweat, we next examined whether sweat stimulates signal activation and induces gene expression. To characterize sweat-mediated signaling in keratinocytes, we stimulated cells with concentrated sweat obtained from three healthy volunteers, and examined the phosphorylation of IκBα, p38, ERK, JNK, STAT1, and STAT3 by Western blotting. As shown in Fig. 2A, all three sweat samples induced the phosphorylation of IκBα, ERK and JNK after 10∼30 min of stimulation and decreased the level of total IκBα from 10 min, indicating the activation of NF-κB, ERK, and JNK signaling. Stimulation of cells with sweat from volunteer 1 induced weak phosphorylation of p38, whereas the sweat from volunteers 2 and 3 did not activate p38 signaling (data not shown). Moreover, the JAK-STAT signal pathway was not activated by sweat (data not shown).

To evaluate the effect of sweat on the expression of proinflammatory cytokines in human keratinocytes, the mRNA expression of IL-8 and IL-1β was detected by real-time PCR. We observed that all three sweat samples significantly upregulated the mRNA expression of IL-8 and IL-1β in keratinocytes (Fig. 2B). After 4∼8 h of treatment, IL-8 and IL-1β mRNA levels were respectively increased approximately 25∼80 fold and 4∼14 fold (Fig. 2B). Thus, sweat from healthy people directly activated normal human keratinocytes.

### IL-1 in sweat is required for keratinocyte activation and cytokine production

**Figure 3:**
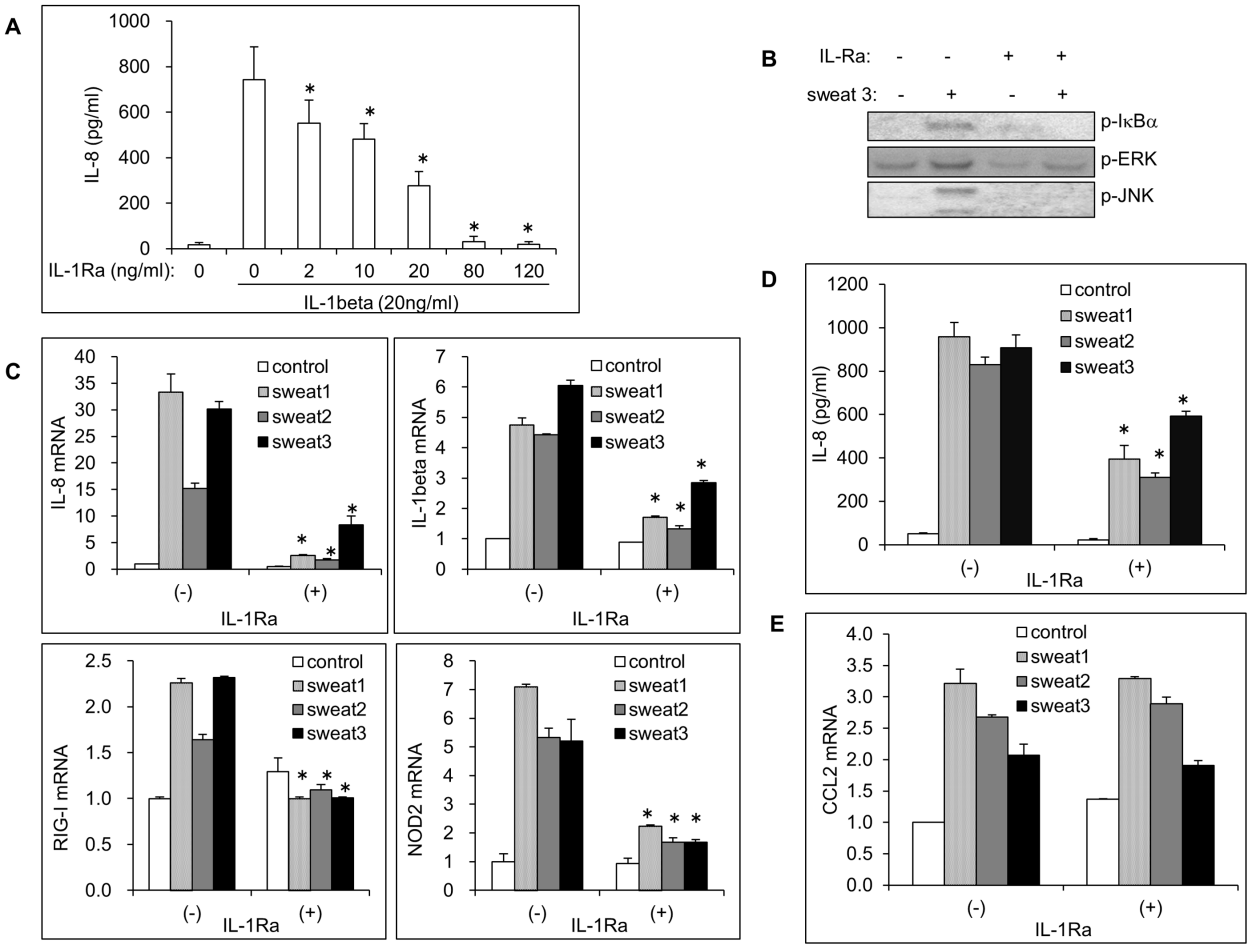
The bioactivity of sweat IL-1 is required for sweat-mediated keratinocyte activation. (A) To block the activation of IL-1 signaling, different concentration of IL-1Ra was applied into cultures for 1 h before stimulation with IL-1β for 24 h, the secretion of IL-8 was detected by ELISA of supernatants. Concentrations represent means±SD (*n* = 3). *p<0.05: compared to that of IL-1β stimulation only. (B) 100 ng/ml IL-1Ra was applied into cultures for 1 h before the addition of concentrated sweat obtained from volunteer 3, keratinocytes were collected after 10 min of stimulation and the phosphorylation of IκBα, ERK and JNK was investigated by Western blotting. Keratinocytes were primed with IL-1Ra for 1 h then stimulated with concentrated sweat obtained from volunteers for 6 h, the mRNA expression of IL-8, IL-1β, RIG-I, NOD2 (C), and CCL2 (E) was detected by real time PCR. Relative mRNA levels were expressed as means±SD (*n* = 3). *p<0.05: compared to that of sweat stimulation without IL-1Ra. (D) Keratinocytes were primed with IL-1Ra for 1h then stimulated with concentrated sweat obtained from volunteers for 24 h, IL-8 secretion from cultures was detected by ELISA of supernatants. Concentrations represent means±SD (*n* = 3). *p<0.05: compared to that of sweat stimulation without IL-1Ra.

Previous studies revealed that sweat-derived IL-1, including both mature and precursor forms of IL-1α and mature IL-β [7], [8], [15], is bioactive and can stimulate the production of IL-6 and IL-8 in cultured human fibroblasts [7]. We further investigated whether the sweat-mediated keratinocyte activation is dependent on the bioactivity of sweat IL-1. IL-1α and IL-1β share a common receptor, IL-1R, and thus have similar biological activities [15]. The bioassay was validated by blocking the IL-1 signal. IL-1Ra, an antagonist of IL-1R, binds to both types of IL-1 receptor (I and II) and competes with IL-1α and IL-1β for receptor binding [15]. As shown in Fig. 3A, pretreatment with IL-1Ra for 1 h suppressed IL-1β -induced IL-8 production in a dose-dependent manner, indicating the blocking of IL-1 signaling by IL-1Ra. To assess the role of IL-1 in the effects of sweat, 100 ng/ml IL-1Ra was added into cultures before sweat treatment. IL-Ra priming completely blocked the phosphorylation of IκBα and JNK, and reduced levels of phospho-ERK, in sweat-treated keratinocytes (Fig. 3B). Moreover, IL-1Ra priming decreased the production of inflammatory cytokines in all three sweat-treated cultures. The induction of IL-8 and IL-1β mRNA was nearly completely blocked (Fig. 3C), and the secretion of IL-8 protein was suppressed by approximately 50∼70% (Fig. 3D). An interesting discovery was that sweat increased the mRNA levels of the innate receptors RIG-I and NOD2 about 2- and 5-fold, respectively, responses that were also inhibited by IL-1Ra (Fig. 3C). However, IL-1Ra failed to affect the induction of CCL2 mRNA by sweat (Fig. 3E). These data suggest that the biological effect of sweat on epidermal keratinocytes is mostly dependent on the bioactivity of sweat IL-1, but that other sweat molecules also contribute to the immune reaction. Considered its high level in eccrine sweat, IL-31 may also contribute to the inflammatory effect of sweat on keratinocytes.

### IL-31 stimulates the activation of keratinocytes, an effect independent of IL-1R

**Figure 4:**
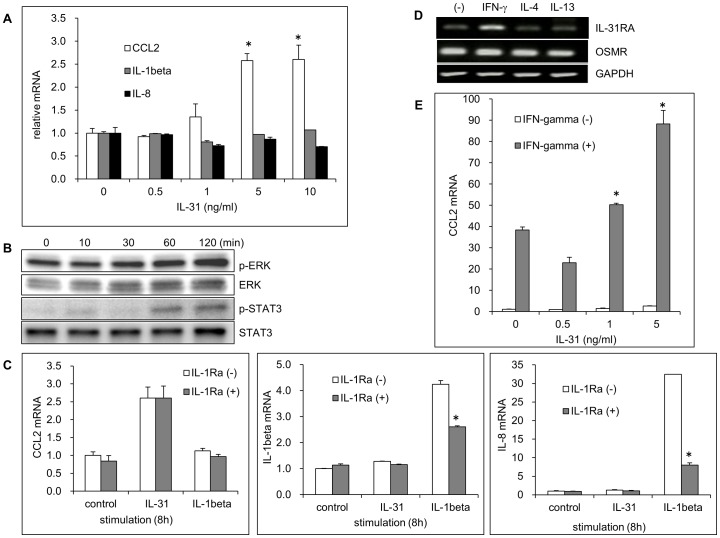
The activation of IL-31 signal in keratinocytes. (A) Keratinocytes were treated with IL-31 (0∼10 ng/ml) for 8 h, the mRNA expression of CCL2, IL-8 and IL-1β were examined by real time PCR. Relative mRNA levels are expressed as means±SD (*n* = 3). *p<0.05: compared to that of control. (B) Keratinocytes were treated with IL-31 (10 ng/ml) for the indicated times, the levels of phospho-ERK, ERK, phospho-STAT3 and STAT3 were decided by western blotting. (The bands for phospho-STAT1 and phospho-AKT were not detected) (C) Keratinocytes were primed with IL-1Ra for 1h before stimulated with IL-31 (10 ng/ml) or IL-1α (10 ng/ml) for 8 h, the mRNA expression of CCL2, IL-1β and IL-8 was detected by real time PCR of total RNA and their relative mRNA levels were expressed as means±SD (*n* = 3). *p<0.05: compared to that without IL-1Ra (D) Keratinocytes were treated with IFN-γ (100 mg/ml), IL-4 (10 ng/ml), or IL-13 (50 ng/ml) for 6 h, the mRNA expression of IL-31RA and OSMR was examined by RT-PCR. (E) Keratinocytes were pretreated with IFN-γ for 24 h, then stimulated with or without IL-31 for 8 h, the mRNA expression of CCL2 were examined by real time PCR. Relative mRNA levels are expressed as means SD (*n* = 3). *p<0.05: compared to that of IFN-γ stimulation only.

Previous reports have showed that extraneous IL-31can activate ERK, AKT, and STATs signal pathways to induce the production of various chemicals in epithelial cells [14]. Both the IL-31 receptors, IL-31RA and OSMR, have been identified in epidermal keratinocytes [14], however, it is confused and not completely clear whether IL-31 directly activates primary keratinocytes_ENREF_18. One recent report argued that recombinant IL-31, at a concentration of 10 ng/ml, could not stimulate keratinocytes, but induced CCL2 expression in keratinocytes pretreated with IFN-γ and pathogenic molecules, which increased cellular expression of IL-31RA [16]. However, we found that stimulation of keratinocytes with IL-31for 8 h increased the mRNA expression of CCL2 (but not that of IL-8 and IL-1β) in a dose-dependent manner, with 5∼10 ng/ml IL-31 increasing CCL2 mRNA levels more than 2.5-fold (Fig. 4A); and IL-31 also induced the rapid phosphorylation of ERK and STAT3 but not AKT (Fig. 4B), suggesting that IL-31activates the ERK and JAK-STAT3 signal pathways in keratinocytes. This difference may be due to the variations in cells and experimental conditions used. We next investigated whether IL-31 activation of keratinocytes is related to IL-1R. In contrary to IL-31, IL-1 induced the expression of IL-8 and IL-1β but not CCL2 in keratinocytes (Fig. 4C). Though IL-1Ra priming significantly suppressed the activation of IL-1 signal in keratinocytes, it could not affect the induction of CCL2 mRNA by IL-31. These data suggested that the activation of IL-31signal in primary keratinocytes is not related to IL-1R.

Confirmed the previous report [16], we found that Th1 cytokine IFN-γ, which is involved in the maintenance of chronic AD, but not Th2 cytokines IL-4 and IL-13, which are predominant in the acute phase of AD, significantly induced the mRNA expression of IL-31RA in keratinocytes (Fig. 4C). IFN-γ is also a strong inducer of CCL2 expression, and IL-31 stimulation following IFN-γ priming resulted in the great synergistic induction of CCL2 mRNA in keratinocytes (Fig. 4D). The synergistic effect of IFN-γ and IL-31 on the activation of keratinocytes might result from the increasing expression of IL-31RA.

## Discussion

We confirmed the presence of IL-1α and IL-1β in sweat from healthy volunteers, and demonstrated that sweat directly activate keratinocytes to produce inflammatory cytokines, a response that mainly depends on the bioactivity of sweat IL-1. In addition, we demonstrated for the first time the *in situ* expression of IL-31 in sweat eccrine glands, detected large amounts of IL-31 in sweat samples, and presented the specific inflammatory effect of IL-31 on primary keratinocytes.

Although all the proinflammatory cytokines that can be detected in eccrine glands (IL-1α, IL-1β, IL-6, TNF-α, and IL-8) were quantified in sweat patches [17] by recycling immunoaffinity chromatography, a highly sensitive methodology, immunoassay of collected sweat samples only detected IL-1α, IL-1β, and IL-31 in our study. IL-1β is easily detectable in sweat from palms and soles, but not in sweat collected from the trunk (IL-1α/β ratio >700 in the trunk and 5.4 in the palms and soles) [8]. Levels of IL-1 in sweat collected from arms (IL-1α/β ratio ∼4) and from the trunk are lower than those in sweat from the palms and soles, skin regions with high densities of eccrine glands. IL-8 can be quantified by ELISA in sweat from the trunk and palms[6], but is hardly detected in sweat obtained from the arms of most volunteers. These data suggest a site-dependent difference in the sweat excretion of cytokines.

The precise cellular source of sweat cytokines is not clear, despite their expression in eccrine glands [11], [13]. Sweating plays a role in the clearance of waste products. Circulating cells express both IL-1 and IL-31, and serum IL-1 and IL-31 can be detected even in healthy people [17], [18]. Cytokines levels in sweat produced spontaneously, without any stimulation or stress, closely correlate with their plasma levels [17], suggesting that a portion of sweat cytokines come from the plasma. However, the IL-1 concentration, which is low in spontaneously produced sweat, is much increased during exercise and in the sauna. This does not appear to represent an excretory process aimed at clearing blood IL-1, but rather indicates a stress-induced increased secretion of IL-1 by eccrine gland cells [8]. Sweat molecules can be derived from eccrine gland cells, or epidermal cells, depending on the sweat collection method used. The collection of sweat over an oil barrier placed on the skin can eliminate contamination by epidermal proteins [7]. We found that the concentrations of IL-1β and IL-31 in sweat collected over an oil barrier were slightly decreased (data not shown). Therefore, the sweat IL-1β and IL-31 detected in our study are partially epidermal. Both forms of IL-1 are distributed throughout the eccrine gland apparatus [12], [13], and are secreted from secretory cells after excise [7]. Similar to sweat IL-1, IL-31 can be detected in the epithelium of both eccrine glands and ducts, but with prominent expression in the epithelial cells of eccrine ducts. It is not clear which stimuli can cause the release of cytokines into sweat. Environmental pathogens and allergens, which can stimulate the secretion of IL-1α, IL-1β [9], [19], [20], and IL-31 (unpublished data) from epidermal keratinocytes, could penetrate deep into some eccrine glands under stress conditions and directly stimulate the epithelium of the gland and duct to secrete IL-1 and IL-31. Regardless of their cellular source, sweat cytokines secreted from sweat gland cells, together with some epidermal proteins, will penetrate the skin tissue to irritate epidermal cells when the skin barrier is defective.

In the present study, we demonstrated that sweat activates the NF-κB, ERK, and JNK signaling pathways and induces IL-8, IL-1β, NOD2, and RIG-I in epidermal keratinocytes, responses dependent on the bioactivity of sweat IL-1. Therefore, sweat IL-1 can activate both dermal fibroblasts [7] and epidermal keratinocytes. Sweat significantly stimulates the production of IL-8, a cytokine that can recruit neutrophils. Several reports show that IL-8 is essential for recruiting neutrophils to the lesional skin in palmoplantar pustulosis [21], [22]. It seems that lesional or systemic IL-8 increases may contribute to the pathogenesis of pustular skin lesions of neutrophilic dermatosis. Induction of the innate receptors NOD2 and RIG-I by sweat IL-1 indicates the possible contribution of sweat to pathogen molecule-mediated skin inflammation.

Itching is the major manifestation of AD and other chronic skin disorders, and exacerbated by stimuli that induce sweating, an effect that may be related to the high levels of IL-31 in sweat. IL-31 is associated with various pruritic skin diseases [14] and was recently identified as a major pruritic factor in AD [23], [24]. It can induce itching by stimulating skin nerve endings, or by activating epidermal keratinocytes to produce certain chemicals [14]. We here demonstrated that extraneous IL-31 directly activates primary keratinocytes *in vitro*: stimulating the activation of ERK and STAT3 signal pathways, and inducing cytokine expression. In addition to IL-8 and IL-1β, sweat induced the expression of CCL2 mRNA, a response that was not inhibited by IL-1Ra, suggesting that CCL2 induction is not due to sweat IL-1, but may instead be due to sweat IL-31, as extraneous IL-31 but not IL-1 specifically induces the expression of CCL2 in primary keratinocytes. Sweat IL-31 seems to induce CCL2 expression by activating ERK and STAT3 pathways. Although we failed to detect the significant phosphorylation of STAT3 in sweat-treated keratinocytes, we could not ignore the possible inflammatory effect of sweat IL-31 on epidermal keratinocytes, specifically in some skin disorders, such as AD, as IL-31RA expression is relatively elevated in AD lesions [14].

In addition to healthy volunteers, we also collected sweats from 3 patients with AD. We detected similar levels of IL-1α and IL-31, but much higher levels of IL-1β in sweat of AD patients (data not shown). On account of the limited number of sweat samples obtained from AD patients, we failed to get a conclusion about whether the sweat of AD patients contains higher levels of cytokines than that of healthy people through a statistic analysis. In spite of these, we could not ignore the irritant role of sweat for the skin of AD patients. Sweat is secreted onto the skin's surface and does not come into contact with keratinocytes in normal skin. However, in skin with a defective cutaneous barrier, such as atopic dermatitis-affected skin, or with rupture of intra-epidermal eccrine ducts, as occurs in miliaria, sweat cytokines, such as IL-1 and IL-31, can directly activate epidermal keratinocytes, inducing the production of inflammatory cytokines and other immune-related molecules to stimulate or exacerbate skin inflammation. Some proinflammatory cytokines affect skin barrier that can further enhance their *in situ* inflammatory stimulation on epidermal keratinocytes. In the AD lesions, the IL-31 released together with sweat may contribute to this vicious circle by inducing itching behavior [14] and by suppressing filaggrin expression and thereby interfering with epidermal differentiation [25] to impair skin barrier. In addition to cytokines, sweat-derived antimicrobial peptides may also contribute to the cutaneous immunity by activating keratinocytes [5], [26].

In conclusion, we detected large amounts of IL-1α, IL-1β, and IL-31 in sweat obtained from healthy volunteers, and demonstrated that sweat can directly activate epidermal keratinocytes, as a danger signal. Our findings suggest that sweat cytokines, notably IL-1 and IL-31, might play roles in the aggravation of some sweat-related skin disorders, such as AD and miliaria rubra, by triggering immune reactions in epidermal keratinocytes.
